# Continuous Apomorphine Infusion in Multiple System Atrophy Real‐World Insights From a French Nationwide Retrospective Cohort

**DOI:** 10.1002/mdc3.70644

**Published:** 2026-04-19

**Authors:** Simon Lamy, Frederique Leh, Alexandra Foubert‐Samier, Soizic Leguy Bonnet, Guillaume Baille, Margherita Fabbri, Margaux Dunoyer, Lise Mantisi, Caroline Giordana, Anaïs El Ouartassi, Olivier Rascol, Wassilios G. Meissner, David Bendetowicz, Marie Vanel, David Grabli, Fabienne Ory‐Magne, Sophie Drapier

**Affiliations:** ^1^ Department of Neurology Pontchaillou University Hospital Rennes France; ^2^ Department of Neurology Pontchaillou University Hospital, CIC INSERM 1414 Rennes Rennes France; ^3^ CHU Bordeaux, Service de Neurologie des Maladies Neurodégénératives, IMNc, CRMR AMS Bordeaux France; ^4^ Université de Bordeaux, CNRS, IMN, UMR5293 Bordeaux France; ^5^ Bordeaux Population Health Research Center, Inserm U1219, University of Bordeaux Bordeaux France; ^6^ Cabinet de Neurologie Libérale Saint‐Malo France; ^7^ Department of Neurology Rothschild Foundation Hospital Paris France; ^8^ Department of Clinical Pharmacology and Neurosciences, Toulouse Parkinson Expert Centre, Toulouse NeuroToul Center of Excellence in Neurodegeneration (COEN), French NS‐Park/F‐CRIN Network University of Toulouse 3, CHU of Toulouse, INSERM Toulouse France; ^9^ Toulouse NeuroImaging Center University of Toulouse, Inserm, UPS Toulouse France; ^10^ MSA French Reference Center Univ. Hospital Toulouse Toulouse France; ^11^ Department of Neurology CRMR MSA, Pitié‐Salpêtrière Hospital, APHP Paris France; ^12^ Department of Neurology‐Parkinson's Disease Expert Center Institute of Neurology, AP‐HP, Pitié‐Salpêtrière University Hospital Paris France; ^13^ Hôpitaux de Paris (AP‐HP) Pitié‐Salpêtrière Hospital Paris France; ^14^ Department of Neurology, NS‐PARK/FCRIN Network Centre Hospitalier Universitaire de Nice, Université Côte d'Azur Nice France; ^15^ Neurology Department Centre Hospitalier d'Antibes Antibes France; ^16^ French National Reference Center for MSA, Clinical Investigation Centre CIC1436, Department of Neurosciences and Clinical Pharmacology, and NS‐Park/FCRIN Network University of Toulouse, University Hospital of Toulouse, INSERM Toulouse France; ^17^ Department of Neurology for Neurodegenerative Diseases University Hospital Bordeaux Bordeaux France; ^18^ Institute for Neurodegenerative Diseases CNRS UMR 5293, University Bordeaux Bordeaux France; ^19^ Cabinet de neurologie Cesson Sévigné France; ^20^ Sorbonne Université, Paris Brain Institute, Inserm, CNRS Paris France; ^21^ Department of Neurology Pitié‐Salpêtrière Hospital, APHP Paris France; ^22^ Department of Neurology, Toulouse Parkinson Expert Centre INSERM, UMR1214 Toulouse NeuroImaging Centre TONIC, University Hospital of Toulouse Toulouse France

**Keywords:** apomorphine, fluctuations, infusion, MSA, parkinsonism

## Abstract

**Background:**

Continuous subcutaneous apomorphine infusion (CSAI) is effective in Parkinson's disease but has not been evaluated in multiple system atrophy (MSA).

**Objective:**

To assess the 6‐month efficacy and tolerability of CSAI in MSA patients.

**Methods:**

French multicenter retrospective registry‐based analysis of CSAI use in MSA. The primary outcome was the 6‐month clinical response using the Clinical Global Impression Improvement (CGI‐I) scale. Secondary outcomes included tolerability, indications, infusion parameters, and treatment course.

**Results:**

Fifty patients were included; 94% had MSA‐P. All had motor fluctuations and 88% showed a clinically meaningful dopaminergic response. CSAI was initiated for motor symptoms (100%) or pain (54%). At 6 months, most patients improved (36% “much,” 54% “minimally”), mainly due to reduced motor fluctuations. Only 8% discontinued CSAI. Tolerability was rated good/excellent in 74%; 20% reported somnolence and/or orthostatic hypotension.

**Conclusion:**

In selected MSA patients with partial dopaminergic responsiveness and motor fluctuations, CSAI provided short‐term clinical benefit with acceptable tolerability.

Multiple system atrophy (MSA) is a rare, rapidly progressive neurodegenerative disorder characterized by autonomic failure in combination with parkinsonian (MSA‐P) or cerebellar (MSA‐C) features.^1^, ^2^ Therapeutic options remain limited to symptomatic management. Although levodopa responsiveness is generally poor in MSA, a subset of patients may experience clinically meaningful benefit, occasionally accompanied by motor and non‐motor fluctuations.^3^ This raises the question of whether device‐aided therapies (DAT)—widely used in advanced Parkinson's disease (PD)—could be beneficial in selected MSA patients. To date, only isolated reports of DAT use in MSA have been published, mainly involving deep brain stimulation or levodopa‐carbidopa intestinal gel.^4^, ^5^ Continuous subcutaneous apomorphine infusion (CSAI) has not been formally investigated in this population. Despite being off‐label, CSAI has been used in a substantial number of MSA patients in France. This retrospective multicenter study aims to assess the efficacy and tolerability of CSAI in MSA, in order to clarify its potential therapeutic role.

## Methods

This retrospective multicenter registry‐based analysis was approved by the Rennes University Hospital Ethics Committee (approval number 25.169). CSAI were initiated between April 2011 and November 2024. Patients were identified through the French MSA network, including certified reference and competence centers. Eligible patients had a diagnosis of MSA according to the 2008 Gilman revised diagnostic criteria^6^ or the Movement Disorder Society (MDS) MSA criteria.^2^ All diagnoses were retrospectively re‐evaluated by each referring expert center based on the MDS criteria. Only patients with a clinically established or clinically probable diagnosis of MSA who received CSAI were included. The primary objective was to evaluate the clinical efficacy of CSAI at 6 months after initiation. The secondary objectives were to evaluate treatment tolerability, the occurrence of adverse events, and overall clinical outcomes during follow‐up under CSAI.

### Data Collection

Anonymized medical data were collected through direct transmission or local databases. Only limited data for standardized assessment tools (UMSARS or a formal levodopa challenge test) were available and were therefore not considered.

#### Baseline Data Collection

At baseline (ie, at CSAI initiation), the following data were collected: demographic data, MSA subtype, diagnostic certainty, motor symptoms and non‐motor symptoms (notably dysautonomia), treatments, including the levodopa equivalent daily dose (LEDD, calculated according to Tomlinson),^7^ the number of daily levodopa intakes, the use of antihypotensive treatments, the clinical response to levodopa. Levodopa response was categorized into two levels based on declarative patient‐reported outcomes: Significant benefit, described as “response,” “efficacy,” or improvement qualified as “good,” “strong,” or “moderate;” and no or minimal benefit described as “absence,” “poor improvement,” or equivalent terms. Indication for CSAI initiation: categorized into four groups: motor symptoms (including motor fluctuations and refractory parkinsonism, defined as persistent parkinsonian features despite some levodopa responsiveness), pain (including wearing‐off pain and dystonia); swallowing difficulties, and peak‐dose non‐motor adverse effects (such as orthostatic hypotension). Multiple indications could be recorded for the same patient.

#### Six‐Month (M6) Data Collection

Follow‐up data at 6 months (M6) included: Therapeutic information, ie, infusion parameters (dose and schedule), concomitant therapies (total LEDD from both CSAI and oral treatments) and daily number of levodopa intakes. Efficacy was retrospectively assessed by using the Clinical Global Impression Improvement (CGI‐I) scale (1 = Very much improved, 7 = Very much worse).^8^ Assessments were performed either by the original participating center or by the neurology team at Rennes University Hospital based on clinical record review. For patients reporting improvement or worsening, the specific symptoms were documented; multiple improvements could be recorded for a single patient.

All adverse effects were recorded. Overall tolerability was assessed using a 5‐point ordinal qualitative global rating scale, specifically designed for the study, integrating the severity of adverse effects, their impact on daily life, and treatment discontinuation, and consistent with commonly used clinician‐rated global assessment methods in pharmacological studies using explicitly defined ordinal categories: 1 = Excellent: explicitly described as excellent, with no adverse effects. 2 = Good: explicitly described as “good” or considered well tolerated, with no adverse effects or only minor ones without daily impact. 3 = Moderate: at least one adverse effect significantly impacting daily life. 4 = Poor: one or more intense adverse effects without CSAI discontinuation. 5 = Very poor: adverse effects severe enough to lead to CSAI discontinuation. CSAI discontinuations within the first 6 months was recorded.

#### Long‐Term Follow‐Up Data Collection

Long‐term follow‐up was assessed using the most recent available data as of May 2025. These included: duration of follow‐up from CSAI initiation until loss to follow‐up or death; total CSAI treatment duration and the number of treatment discontinuations.

### Statistical Analyses

Descriptive statistics are presented as median (range) for continuous variables, and as count (percentage) for categorical variables. All statistical analyses were conducted using GraphPad Prism version 10 (GraphPad Software, La Jolla, CA, USA).

## Results

### Baseline Patient Characteristics and Indication for CSAI Use

Fifty patients with MSA treated with CSAI were included. Thirty‐three (66%) met criteria for clinically established MSA. Median age at CSAI initiation was 59.9 years and median disease duration was 5.4 years. Most patients had MSA‐P (94%). All patients exhibited motor fluctuations, and 88% showed a clinically meaningful response to dopaminergic therapy. The median LEDD was 1075 mg/day (six intakes per day). Orthostatic hypotension was documented in 66% of cases. The primary indication for CSAI was the management of motor symptoms, reported in all patients (100%). In 54% of cases, CSAI was also initiated for pain management. All characteristics and indications are detailed in Table 1.

**TABLE 1:** Baseline characteristics of the study population and CSAI indications (N = 50)

Variable	Value	(%) or Range
Sex: Female	32	(64.0)
MSA‐P subtype	47	(94.0)
Diagnostic certainty		
Clinically established MSA	33	(66.0)
Clinically probable MSA	17	(34.0)
Age (years)^a^		
At symptom onset	53.7	(41.1–68.1)
At diagnosis	58.7	(46.4–70.0)
At CSAI initiation	59.9	(47.5–73.0)
Disease duration (years)^a^	5.4	(2.2–18.9)
Motor and non‐motor symptoms		
Parkinsonism	50	(100.0)
Tremor	31	(62.0)
Motor fluctuations	50	(100.0)
Wearing‐off	50	(100.0)
Peak‐dose dyskinesia	18	(36.0)
Gait difficulties	50	(100.0)
Cerebellar signs	33	(66.0)
Falls	39	(78.0)
Use of walking aid	38	(76.0)
Walking stick	11	(28.9)
Walker/rollator	22	(57.9)
Wheelchair	5	(13.1)
Orthostatic hypotension	33	(66.0)
Lower urinary tract symptoms	45	(90.0)
Erectile dysfunction (among males)	16/18	(88.9)
Pyramidal signs	32	(64.0)
Dysarthria	42	(84.0)
Cognitive impairment (MoCA <25)	10	(20.0)
Response to levodopa		
Significant benefice	44	(88.0)
No or minimal response	6	(12.0)
Treatment at baseline (before CSAI initiation)		
Levodopa equivalent daily dose (mg)^a^	1075	(300–2212)
Daily number of L‐Dopa intakes, including extended‐release formulations^a^	6	(3–10)
Dopamine agonist	11	(22.0)
Midodrine or fludrocortisone	13	(26.0)
Indication of CSAI initiation		
Motor symptoms	50	(100.0)
Refractory parkinsonism	46	(92.0)
Motor fluctuations	42	(84.0)
Pain	27	(54.0)
Painful dystonia	21	(42.0)
Wearing‐off pain	21	(42.0)
Swallowing difficulties	7	(14.0)
Peak‐dose oral levodopa non‐motor adverse effects (eg, orthostatic hypotension, diarrhea)	4	(8.0)

^a^ Values are presented as median and range (minimum–maximum).

### Six‐Month Outcomes

At 6 months, most patients reported much (36%) or minimally (54%) clinical improvement. Benefits were mainly related to reduced motor fluctuations. Among patients treated for pain, most reported pain improvement. CSAI was generally well tolerated. Somnolence and/or orthostatic hypotension occurred in 20%. Only four patients (8%) discontinued CSAI within 6 months. Six‐month infusion parameters and outcomes are detailed in Table 2.

**TABLE 2:** CSAI parameters and clinical efficacy/tolerability at 6‐month follow‐up (M6)

Parameter	Value	(%) or range
Infusion modality		
Daytime only	20	(40.0)
Nighttime only	0	(0.0)
Continuous (24 h a day)	30	(60.0)
Flow rate (mg/h)^a^		
Daytime	3.7	(1.6–8.2)
Nighttime	2.1	(0.5–5.0)
Infusion duration (hours)^a^		
Daytime	15	(12–18)
Nighttime	9	(6–12)
LEDD (mg/day)^a^		
Total	1619	(565–2700)
From CSAI	619	(260–1330)
From oral therapy	1000	(60–1700)
Daily number of L‐Dopa intakes, including extended‐release formulations	6	(1–11)
CGI global rating		
1 – Very much improved	0	(0.0)
2 – Much improved	18	(36.0)
3 – Minimally improved	27	(54.0)
4 – No change	4	(8.0)
5 – Minimally worse	1	(2.0)
6 – Much worse	0	(0.0)
7 – Very much worse	0	(0.0)
Reported clinical benefit		
Reduction in motor fluctuations	40	(80.0)
Reduction in parkinsonism	32	(64.0)
Reduction in pain (among those with pain indication: n = 27)	22	(81.4)
Reduction in dystonia	18	(36.0)
Improved gait	18	(36.0)
Improved speech	3	(6.0)
Reduced hypersalivation	2	(4.0)
Improved orthostatic hypotension (oral levodopa peak)	1	(2.0)
Tolerability score (1 = Excellent; 5 = very poor)		
1 – Excellent	5	(10.0)
2 – Good	32	(64.0)
3 – Moderate	10	(20.0)
4 – Poor	1	(2.0)
5 – Very poor	2	(4.0)
Side effect		
Daytime drowsiness (new/worsened)	10	(20.0)
Orthostatic hypotension (new/worsened)	10	(20.0)
Worsening of hypotension in patients on antihypotensives (n = 13)	1	(4.0)
Subcutaneous nodules	8	(16.0)
Nausea or vomiting	4	(8.0)
Visual hallucinations	4	(8.0)
Impulse control disorders	3	(6.0)
Dyskinesias	2	(4.0)
Local allergic reactions	1	(2.0)
Peripheral edema (lower limbs)	1	(2.0)
CSAI discontinuation within 6 months	4	(8.0)

^a^ Values are presented as median and range (minimum–maximum).

### Follow‐Up

The median follow‐up duration from CSAI initiation to last contact or death was 2.0 years (range: 0.5–12.5). During this period, 11 patients (22%) discontinued CSAI, after a median treatment duration of 10.7 months (range: 0.3–42.8 months) and were subsequently switched back to optimized oral dopaminergic therapy. Although reasons for discontinuation were not consistently documented, disease progression or changing perceived benefit–risk balance were likely contributing factors. By the end of follow‐up, 20 patients (40%) had died.

## Discussion

The management of atypical parkinsonian syndromes remains a major challenge owing to limited therapeutic options. CSAI is well‐established in PD, where it reduces motor fluctuations,^9^, ^10^ improves quality of sleep and life,^11^, ^12^ and in some cases, it could help alleviate pain and improve comfort in end‐of‐life palliative situations.^13^, ^14^, ^15^, ^16^ In France, CSAI is administered through a structured care model involving specialized home‐care providers. This organizational structure supports the safe, long‐term use of CSAI,^17^, ^18^ even for indications that may fall outside conventional consensus guidelines (eg, APO^2^).^19^

As a result, some clinical teams have started exploring CSAI in patients with MSA presenting with motor fluctuations or dopaminergic‐responsive symptoms inadequately controlled by oral therapies. Although patients with MSA typically show a limited or absent response to dopaminergic therapy,^2^ a subset of patients may nevertheless exhibit a clinically meaningful response to levodopa. In the European MSA cohort,^3^ 31.2% of patients experienced a transient clinical benefit from levodopa, and motor fluctuations were reported in some cases, with end‐of‐dose wearing‐off observed in 23% of patients. A recent literature review also reported a beneficial response to levodopa in 35.9% of 293 pathologically confirmed MSA cases. ^20^ These findings suggest that only a selected subgroup of patients may be candidates for DAT. While a few case reports have suggested that levodopa–carbidopa intestinal gel (LCIG) may offer potential benefits in MSA,^5^ to date no systematic investigation of CSAI has been conducted in this population.

Over a 14‐year period, we identified only 50 patients with MSA treated with CSAI. This cohort represents a selected population with a phenotype distinct from that described in the European MSA cohort.^3^ Notably, this cohort showed a higher prevalence of the parkinsonian subtype (94% vs. 61.7%), greater dopaminergic responsiveness (88% vs. 31.2%), and universal presence of motor fluctuations. These characteristics motivated the use of CSAI in this population.

The short‐term benefit–risk profile of CSAI appeared favorable in this carefully selected MSA cohort. The six‐month discontinuation rate was low (8%) and compared favorably with rates reported in Parkinson's disease studies, including TOLEDO (22.6% at 3 months) and InfusON (30.3%).^10^, ^21^ Most patients reported clinical improvements, particularly reduced motor fluctuations. Some patients improved dystonia‐related pain, consistent with findings in other atypical parkinsonian syndromes such as progressive supranuclear palsy and corticobasal syndrome.^22^

CSAI was generally well tolerated. While 20% of patients experienced increased somnolence or orthostatic hypotension, no discontinuations were attributed to these side effects. These findings align with PD data (eg, Meira et al reported 22.9% orthostatic hypotension and 30.6% somnolence after 6 months).^23^ The observed tolerability may also reflect a higher acceptance threshold in a population with limited therapeutic options.

In many respects, the therapeutic rationale in this cohort resembled that of the “late‐pump” profile described in PD and characterized by moderate dopaminergic responsiveness, high oral dosing frequency, complex symptomatology (axial symptoms, dysautonomia) and infusion rates around 3–4 mg/h.^19^ CSAI was systematically used as an adjunct to oral dopaminergic therapy, with a 46% median increase in total LEDD, while oral medication was typically maintained. Although an increase in oral dopaminergic therapy could have been considered, the presence of motor fluctuations—similar to those observed in PD—supported the rationale for continuous dopaminergic infusion. Notably, dose escalation of the continuous therapy was what ultimately stabilized the patient.

This study has several strengths, including its multicenter, nationwide data collection and the relatively large sample size for a rare disease. Diagnostic validation using MDS criteria and confirmation by expert centers enhances the reliability of the findings. The main limitations relate to its retrospective design and the lack of standardized rating scales which limit objective assessment of efficacy and tolerability.

In conclusion, this retrospective multicenter registry‐based analysis suggests that CSAI may offer an effective and potentially safe therapeutic benefit for selected MSA patients with partial dopaminergic responsiveness and motor fluctuations. In a population marked by limited treatment alternatives and high unmet needs, even mild improvements in motor symptoms, pain, and quality of life can be clinically meaningful. These findings underscore the urgent need for prospective, controlled studies to delineate the true potential—and limitations—of this intervention in atypical parkinsonian syndromes.

## Author Roles

(1) Research project: A. Conception, B. Organization, C. Execution; (2) Statistical Analysis: A. Design, B. Execution, C. Review and Critique; (3) Manuscript Preparation: A. Writing of the first draft, B. Review and Critique.

S.L.: 1A, 1B, 1C, 2A, 2B, 3A, 3B.

F.L.: 1B, 1C, 3B.

A.F.S.: 1B, 1C, 3B.

S.L.B.: 1B, 1C, 3B.

G.B.: 1C, 3B.

M.F.: 1C, 3B.

M.D.: 1C, 3B.

L.M.: 1C, 3B.

C.G.: 1C, 3B.

A.E.O: 1C, 3B.

O.R.: 1C, 3B.

W.G.M.: 1C, 3B.

D.B.: 1C, 3B.

M.V.: 1C, 3B.

D.G.: 1C, 3B.

F.O.M.: 1C, 3B.

S.D.: 1A, 1B, 1C, 2C, 3B.

## Disclosures

**Ethical Compliance Statement:** This study was approved by the Ethics Committee of Rennes University hospital (approval number 25.169). Data were retrospectively collected from anonymized routine medical charts. Informed consent was obtained through an opt‐out method, with a letter of non‐opposition sent to all patients across participating centers. We confirm that we have read the Journal's position on issues involved in ethical publication and affirm that this work is consistent with those guidelines. All authors have read and complied with the Journal's Ethical Publication Guidelines. “We confirm that we have read the Journal's position on issues involved in ethical publication and affirm that this work is consistent with those guidelines.”

**Funding Sources and Conflicts of Interest:** No specific Funding was received for this work. The authors declare that there are no conflicts of interest relevant to this work.

**Financial Disclosures for the Previous 12 Months:** SL reports travel funding from Elivie. FL reports travel funding from Elivie. AFS reports received honoraria from Aguettant Laboratory and Abbvie and Sanofi, travel support received from ELivie and grants from the French Rare Disease Foundation, from the French regional health agency (Agence Régionale de Santé de nouvelle Aquitaine), France Parkinson association and Fayat Foundation. SLB declares no disclosures. GB received honoraria to speak from ABBVie, Orkyn, NHC, ELIVIE, Abbott. Consultancies for ELIVIE, ABBvie, Aguettant. MF received honoraria to speak from ABBVie, Orkyn, Everpharma and BIAL. Consultancies for Medtronic and Teitur Throphics. MD received honoraria for speeches from Novartis. Travel funding received from Merz, Elivie. LM served on scientific advisory boards for Abbvie, received honoraria for speeches from NHC, Elivie, Novartis, Orkyn (Air Liquide), received travel funding from Orkyn (Air Liquide). CG received honoraria from Abbott Médical. Consultancies for Orion Pharma. AEO declares no disclosures. OR has no direct conflict of interest with the present study. He received honorarium for scientific advise or consultancy from Apopharma, Astrazeneca, Bial, Biogen, Britannia, Cerevel, Contera, GE Healthcare, Ionis, LGD Nuvamid, Lundbeck, Merz, Neuralight, Neuroderm, ONO Pharma, Orion Pharma, PD Neurotechnology, Roche Therapeutics, Sanofi, Servier, Sunovion, Supernus, Thelonius Mind, Takeda, Teva, UCB, Zambon and has received scientific grants from Agence Nationale de la Recherche (ANR), CHU de Toulouse, France‐Parkinson, Michael J. Fox Foundation, French Ministry of Health (Programme Hospitalier de Recherche Clinique), European Commission (H2020 & Horizon Europe), CURE PARKINSON; ONO pharma and Lundbeck. WGM is unrelated to the present work. He has received honoraria for consultancy from Lundbeck, Inhibikase, Tree Frog, Koneksa, Takeda, and Ferrer. He has also received lecturing fees from Lundbeck. DB received honoraria from Orkyn, travel support from CHU de Bordeaux and a grant from Fayat Foundation. MV declares no disclosures. DG received grants from AP‐HP (DRC‐PHRC), received research funding from Air Liquide, Merz and NHC; speech honorarium from NHC, Elivie, Medtronic, Merz and Orkyn; received travel funding from Abbvie and Merz. FOM has served as an advisory board member or consultant or has received travel grants from AbbVie, Aguettant, Orphalan, Orkyn, Biogen, Adelia, NHC France, Convatec. She reports grants from Fondation Santé Service. SD has served as an advisory board member or consultant or has received travel grants from AbbVie, Elivie, Medtronic, Orkyn, Sanofi, Teva.

## Financial Disclosures and Conflicts of Interest

Author disclosures are available in the Supporting Information.

## Supporting information


**Data S1.** Coi_disclosure.

## Data Availability

The data that support the findings of this study are available from the corresponding author upon reasonable request.
